# Identifying Patterns of Clinical Interest in Clinicians’ Treatment Preferences: Hypothesis-free Data Science Approach to Prioritizing Prescribing Outliers for Clinical Review

**DOI:** 10.2196/41200

**Published:** 2022-12-20

**Authors:** Brian MacKenna, Helen J Curtis, Lisa E M Hopcroft, Alex J Walker, Richard Croker, Orla Macdonald, Stephen J W Evans, Peter Inglesby, David Evans, Jessica Morley, Sebastian C J Bacon, Ben Goldacre

**Affiliations:** 1 Bennett Institute for Applied Data Science Nuffield Department of Primary Care Health Sciences University of Oxford Oxford United Kingdom; 2 Department of Medical Statistics London School of Hygiene and Tropical Medicine London United Kingdom

**Keywords:** prescribing, NHS England, antipsychotics, promazine hydrochloride, pericyazine, clinical audit, data science

## Abstract

**Background:**

Data analysis is used to identify signals suggestive of variation in treatment choice or clinical outcome. Analyses to date have generally focused on a hypothesis-driven approach.

**Objective:**

This study aimed to develop a hypothesis-free approach to identify unusual prescribing behavior in primary care data. We aimed to apply this methodology to a national data set in a cross-sectional study to identify chemicals with significant variation in use across Clinical Commissioning Groups (CCGs) for further clinical review, thereby demonstrating proof of concept for prioritization approaches.

**Methods:**

Here we report a new data-driven approach to identify unusual prescribing behaviour in primary care data. This approach first applies a set of filtering steps to identify chemicals with prescribing rate distributions likely to contain outliers, then applies two ranking approaches to identify the most extreme outliers amongst those candidates. This methodology has been applied to three months of national prescribing data (June-August 2017).

**Results:**

Our methodology provides rankings for all chemicals by administrative region. We provide illustrative results for 2 antipsychotic drugs of particular clinical interest: promazine hydrochloride and pericyazine, which rank highly by outlier metrics. Specifically, our method identifies that, while promazine hydrochloride and pericyazine are barely used by most clinicians (with national prescribing rates of 11.1 and 6.2 per 1000 antipsychotic prescriptions, respectively), they make up a substantial proportion of antipsychotic prescribing in 2 small geographic regions in England during the study period (with maximum regional prescribing rates of 298.7 and 241.1 per 1000 antipsychotic prescriptions, respectively).

**Conclusions:**

Our hypothesis-free approach is able to identify candidates for audit and review in clinical practice. To illustrate this, we provide 2 examples of 2 very unusual antipsychotics used disproportionately in 2 small geographic areas of England.

## Introduction

Since 2011, the National Health Service (NHS) in England has openly shared detailed monthly general practice prescribing data to the level of individual doses, chemicals, and brands, aggregated at the individual general practice level. These data have supported original research on a broad range of topics as well as supporting systematic audit and review programs to realize improvements in primary care prescribing [1].

Our group produces OpenPrescribing.net [2], a free and widely used tool where anyone can explore the prescriptions dispensed at any practice in England and monitor prescribing patterns down to the level of individual brands, formulations, and doses. OpenPrescribing offers data-driven feedback to assist regional- and practice-level medicines optimization teams and identifies areas for review of which they may not otherwise have been aware. For example, we identify whether each NHS organization is an outlier on more than 80 predefined measures covering a range of prescribing safety, cost-effectiveness, and efficacy issues. Unique savings opportunities for each practice by making comparisons between brands or generics and formulations are also calculated [3], and there is evidence that these savings are realized [4].

Typically, data science for service audit and quality improvement is hypothesis driven: identifying a targeted behavior and using data to measure the achievement of that goal [5,6]. Given the vast scale of openly available NHS prescribing data (more than 2 billion rows of data covering 8000 organizations during the past decade) and the vast range of clinical behaviors and potential signals for variation in care that may lie within this data set, we set out to develop new hypothesis-free data science techniques to identify new opportunities for service improvement driven by variation in care.

Our overall analytic aim was to prototype and describe methods to identify previously unknown signals of clinical interest in prescribing data (existing methodology to identify outliers often focuses on financial aspects of prescribing [7-9] or is focused on a specific clinical question [10,11]). We ran a series of internal workshops to develop a short list of data science methods that might be used to identify prescribing behaviors that are unusually distributed across NHS organizations or regions. Here, we briefly report the successful deployment of one such method (ranking chemicals by kurtosis and a ratio between intercentile differences across all chemical-class pairs) and demonstrate how this identified high prescribing of unusual antipsychotics in 2 small regions of England.

## Methods

### Study Design and Data Sources

We conducted a cross-sectional study using open NHS prescribing data on all dispensed products prescribed by general practices in England, June-August 2017, extracted from the OpenPrescribing database. A relatively short 3-month window was chosen, owing to the fact that this work represents a proof of concept. The data set includes, for each practice, product and month of prescribing, the number of items prescribed (equivalent to the number of prescription forms on which each product appeared), and the total quantity (eg, tablets and mL). Practices were grouped by their parent Clinical Commissioning Group (CCG), an NHS administrative region. In England, approximately 7000 NHS general practices were arranged into 207 CCGs in 2017.

### Data Processing

All chemicals prescribed in England were assigned to a “class” of chemicals, using their British National Formulary (BNF) legacy code to identify the chemical’s relevant BNF subparagraph. We limited our search to chemicals in chapters 1-15 of the BNF (1511 prescribed chemicals) to exclude chapters not following a chemical/subparagraph structure, which largely cover nonmedicinal products such as dressings. For each chemical-class pair, the number of items (similar to a prescription in prescribing data) that were prescribed for each chemical was expressed as a proportion of the total items prescribed of all chemicals in its class. These chemical-class proportion values were calculated for each CCG. To avoid including rarely prescribed classes of chemicals that would generate spurious findings, we excluded 116 chemicals with the lowest total items prescribed (specifically, the lowest two centiles) and 4 chemicals that were used by less than 50 CCGs. In total, then, 1395 chemicals were subject to analysis.

### Ranking Chemical-Class Pairs by Outlier Metrics

We first sought to focus our analysis on those chemicals with the distribution characteristics indicative of (1) reasonable variability and (2) positive outliers among CCGs (ie, outliers with higher prescribing rates rather than outliers at lower prescribing rates): chemical-class pairs were filtered where range>10% and skew>0. This identified 412 candidate chemicals of interest. To further refine this group of chemicals, we retained only those candidates for which (1) the median proportion was <0.1, that is, those prescribed at a very low rate, or not at all, by most CCGs and (2) the number of prescriptions nationally was not small (at least 1000 prescriptions), so as to limit the impact of random fluctuations in small numbers of prescriptions. These further filtering steps reduced our candidate list to 204 chemicals.

We then implemented 2 alternative ranking approaches to identify outliers among our candidate chemicals. The first was kurtosis, which can be described as a numerical measure of the extent to which the tails of a given distribution are heavier or lighter than a normal distribution; overall, data sets with high kurtosis will tend to have more extreme outliers than data sets with low kurtosis. Kurtosis is a good method for detecting an unknown number of outliers in a data set [12,13]. We calculated the kurtosis for each candidate chemical-class pair across all CCGs and ranked the chemicals by this kurtosis value (highest to lowest). We then generated an alternative ranking of chemicals using a ratio calculated as the intercentile range of the chemical-class proportion between the 95th and 97th centiles (the top prescribing CCGs) to the intercentile range between 50th and 95th centiles (those CCGs prescribing at more moderate rates); this ratio will hereafter be referred to as the “high:mid centile ratio”.

Both approaches sort all chemicals into an order where, for the most highly ranked chemicals, there are very substantial differences between CCGs in the extent to which that chemical is used in the context of all prescribing of all chemicals in its class. This ranking was used to prioritize the chemical-class pairs for manual evaluation by clinical staff (BMK, RC, OM, and BG) for signals of clinical interest.

### Visualizing Prescribing Rates Using Choropleth Maps

For selected chemical-class pairs of clinical interest, we generated a choropleth map using OpenPrescribing.net to visualize the geographical distribution of prescribing of each chemical as a proportion of its class. Data management was performed using Python and Google BigQuery, with the analysis carried out using Python (authors HJC and LEMH). Data and charts, as well as all code for data management and analysis are openly available for inspection and reuse on GitHub [14].

### Ethical Considerations

This study uses exclusively open publicly available data; therefore, no ethical approval was required.

## Results

**Figure 1:**
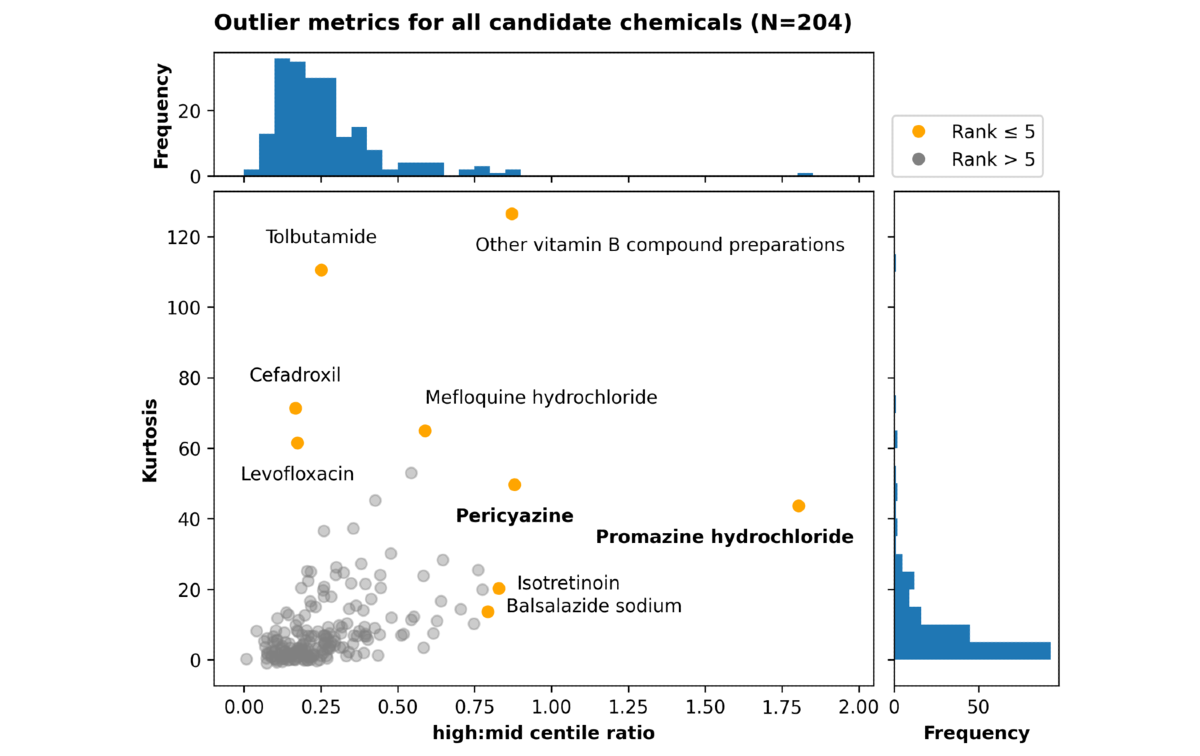
Prioritizing 204 candidate chemicals using ranking by 2 outlier metrics. The top 5 chemicals by either the high:med centile ratio or kurtosis are highlighted in orange; all other chemicals are shown in gray. Each metric is summarized as a histogram of chemical counts along the corresponding axis. Pericyazine and promazine hydrochloride (our chemicals of interest) are highlighted in bold.

A total of 204 chemicals were found to have prescribing rate distributions indicative of positive outliers among administrative regions in the NHS in England. Figure 1 summarizes the high:mid centile ratio and kurtosis value for these chemicals. The top 5 ranked chemicals by either outlier measurement are highlighted.

Clinical review of these results identified 2 chemical substances to illustrate the methodology: promazine hydrochloride (high:mid centile ratio: 1.804, kurtosis: 43.61) and pericyazine (high:mid centile ratio: 0.880, kurtosis: 49.60). These 2 antipsychotic drugs are the top 2 ranking chemicals by high:mid centile ratio and also rank in the top 10 (ninth and seventh, respectively) by kurtosis.

**Figure 2:**
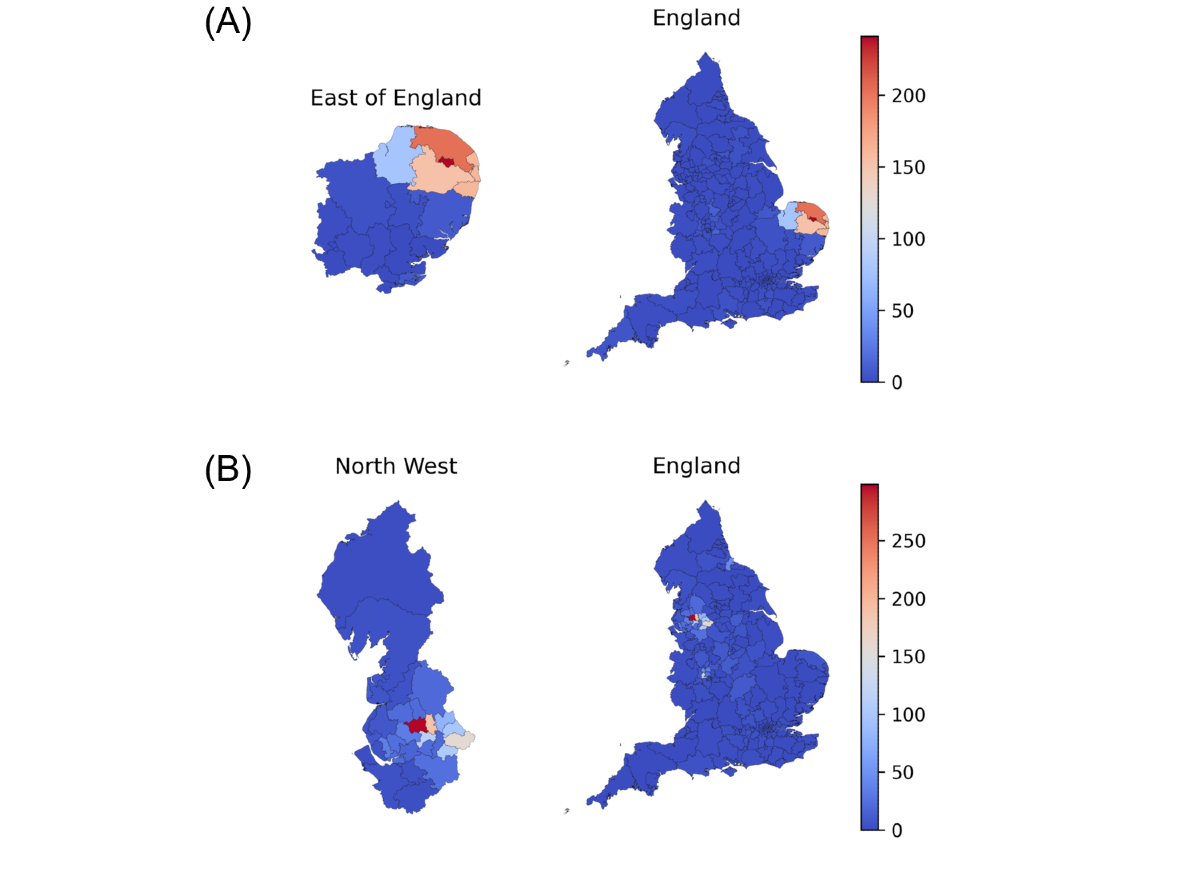
Total number of prescriptions for (A) pericyazine (B) promazine hydrochloride per 1000 antipsychotic prescriptions for all CCGs in England in June-August 2017. The colour scale in each plot indicates the number of prescriptions per 1000 antipsychotic prescriptions in the corresponding geographic region.

Exploring these chemicals in more detail, pericyazine is shown to be prescribed at a much higher rate in the East of England (Table 1), with 13,119 in 277,470 (4.7%) antipsychotic prescriptions being for pericyazine, compared to 15,344 in 2,489,069 (0.6%) nationally. OpenPrescribing choropleth maps demonstrate that this high level of prescribing was concentrated particularly in Norwich and the Norfolk area more widely (Figure 2A; Multimedia Appendix 1). Promazine hydrochloride is shown to be prescribed at higher levels in the North West of England (Table 1), accounting for 20,060 in 412,624 (4.9%) antipsychotic prescriptions compared to 27,724 in 2,489,069 (1.1%) nationally. Again, these outlier prescribing behaviors were concentrated in specific CCGs: Bolton and the wider Greater Manchester area (Figure 2B; Multimedia Appendix 2).

**Table 1:** National and regional prescribing counts and rates (per 1000) of pericyazine (N=15,344) and promazine hydrochloride (N=27,724) in all regions in England (June-August 2017). Prescribing rates for both chemicals are expressed per 1000 antipsychotic prescriptions (N=2,489,069).

Region	Antipsychotics, n	Pericyazine		Promazine hydrochloride	
		Prescribed, n	Per 1000	Prescribed, n	Per 1000
East Midlands	188,593	155	0.8	1088	5.8
East of England	277,470	13,119	47.3	381	1.4
Kent, Surrey, and Sussex	189,490	192	1.0	334	1.8
North Central and East London	151,108	84	0.6	122	0.8
North East	162,212	96	0.6	620	3.8
North West	412,624	315	0.8	20,060	48.6
North West London	89,949	5	0.1	100	1.1
South London	124,296	103	0.8	127	1.0
South West	209,838	306	1.5	131	0.6
Thames Valley	75,489	10	0.1	163	2.2
Wessex	121,442	104	0.9	276	2.3
West Midlands	246,907	580	2.3	3951	16.0
Yorkshire and the Humber	239,651	275	1.1	371	1.5
All	2,489,069	15,344	6.2	27,724	11.1

## Discussion

### Summary

Using a hypothesis-free approach, we have applied data science techniques to a national data set to identify outliers. Following subsequent clinical review, 2 unusual antipsychotic medications, in very limited use nationally, that are very commonly prescribed in 2 small geographic regions of England were identified. Specifically, pericyazine makes up only 0.6% (15,344 of 2,489,069) of all antipsychotic prescriptions nationally, but in Norwich it represents 24.1% (5197 of 21,553) of all antipsychotic prescriptions; promazine hydrochloride makes up 1.1% (27,724 of 2,489,069) of all antipsychotic prescriptions nationally; however, in Bolton, it represents 29.9% (5549 of 18,577) of all antipsychotic prescriptions.

### Strengths and Weaknesses

This study is a proof of concept with a pragmatic and exploratory approach to the methodology and is still under iterative development with regard to optimizing metrics and parameters. As such, we recognize that there are limitations in the metrics that we have used to rank the chemical-class pairs as described here. For example, it is possible that the rankings being generated could be misleading where a small number of CCGs are under prescribers for particular chemicals, thereby inflating the outlier status of the same chemical in other areas. Furthermore, the effect of variability where the number of prescriptions is small is not yet known, although we do seek to mitigate against this by removing chemicals that are prescribed at particularly low volumes. However, we do not present this work as a stand-alone method for outlier detection; rather, we present it as an approach to prioritize and focus on manual clinical audit and review.

Our study does cover a reasonably short period of time (June-August 2017), again owing to it being a proof of concept. However, the OpenPrescribing data set used does include all prescribing in all typical practices in England, thereby minimizing the potential for obtaining a biased sample. Furthermore, the chemicals identified using our approach do represent legitimate targets for further investigation; unfortunately, our reporting of this work and subsequent investigations into the reasons for these prescribing outliers were disrupted by the COVID-19 pandemic.

### Findings in Context

Pericyazine has been used infrequently for schizophrenia and for short-term adjunctive management of severe anxiety, psychomotor agitation, and violent or dangerously impulsive behavior [15]. There is no mention of pericyazine in any guideline on the National Institute for Health and Care Excellence (NICE) website, the main source of clinical guidelines in England. A 2014 Cochrane review on pericyazine identified only 5 studies suitable for inclusion, could not determine the effect of pericyazine in schizophrenia given the low quality of evidence, and found a higher incidence of side effects compared to atypical antipsychotics [16]. A PubMed search identified only 73 publications that contain the word “pericyazine” [17] in any way since 1965 compared with over 22,000 results for haloperidol and 11,000 for risperidone. Promazine hydrochloride is licensed in psychomotor agitation and agitation or restlessness in the older adults [18]. It is not mentioned in any NICE guideline and appears in only 1355 PubMed records [19] (peaking in 1964). We are aware of no prior work using data science techniques hypothesis-free to systematically identify outliers for any given treatment choice or clinical outcome in the manner outlined here.

### Policy Implications and Interpretation

We report only the fact of a substantial deviation from national prescribing norms in these 2 small regions and make no direct comment on the appropriateness of using these medications in any single patient or in general. It was outside the scope of this work to engage in a detailed qualitative or other study to understand the reasons for the high usage of these 2 unusual antipsychotics in these 2 regions; however, we note that promazine hydrochloride and pericyazine have previously appeared in treatment formularies for Greater Manchester and Norfolk, respectively. In addition, it is noted that antipsychotic medication is typically initiated in secondary care, with prescribing taken over in general practice.

The Department of Health and Social Care recently consulted on an ambitious plan to harness data to improve health delivery and outcomes [20]. The use of data to identify variation in clinical activity and outcomes is long established [21,22], and recent flagship projects in the NHS such as RightCare and Getting it Right First Time are focused on identifying and addressing variation in care. However, these approaches typically rely on a traditional approach, whereby desirable clinical activities or outcomes are prospectively defined by clinicians or commissioners, and adherence is then measured by analysing relevant data. It is highly unlikely that these conventional methods would ever have identified the unusual prescribing behaviors reported in this paper. Similarly, it is likely that there are many further clinically interesting signals that could be identified by taking a variety of data-driven approaches to detecting unusual clinical activity or outcomes across the full universe of NHS data.

In our experience of running OpenPrescribing.net, the key barrier to better use of data for service improvement is an unhelpful cultural and practical divide between purely academic work on health data, and practical use of data in service analytics. This is exemplified by, in general, the use of different teams, different funding mechanisms, different institutions, and different data infrastructures. As the methods, data, and overarching objectives of both domains overlap substantially, we hope that funders and commissioners can help to bring these strands of work together.

### Future Research

These findings will contribute to a wider program of work, which aims to develop a range of interactive tools on OpenPrescribing.net to present candidate signals of interest for substantial divergence from national prescribing norms at the level of individual practices, CCGs, and other key NHS organizational groupings such as primary care networks and integrated care systems. For this web-based service, we expect to present a wide variety of signals at scale, without further context on evidence or guidelines, as a trigger for positive local discussion and further exploration by clinical or commissioning teams, and inviting feedback on whether they found the signals to be helpful in identifying any previously unrecognized opportunities to change local prescribing practices or understanding the reasons for any divergences.

### Conclusions

We describe a hypothesis-free approach to identify candidates for audit and review in clinical practice, with examples highlighted of 2 very unusual antipsychotics used disproportionately in 2 small geographic areas of England.

## Appendix

Multimedia Appendix 1 Pericyazine prescriptions in the East of England (June-August 2017).

Multimedia Appendix 2 Promazine hydrochloride prescriptions in the North West of England (June-August 2017).

## Abbreviations

BNF: British National Formulary
CCG: Clinical Commissioning Group
NICE: National Institute for Health and Care Excellence
NHS: National Health Service

## Data Availability

This study uses exclusively open, publicly available data. Processed data is additionally available within the study repository along with all analysis code [14].
